# Prevalence and correlates of subjective cognitive impairment in Chinese psychiatric patients during the fifth wave of COVID-19 in Hong Kong

**DOI:** 10.3389/fpsyt.2023.1216768

**Published:** 2023-08-17

**Authors:** Vivian Shi Cheng Fung, Jacob Man Tik Chan, Eileena Mo Ching Chui, Corine Sau Man Wong, Joe Kwun Nam Chan, Ryan Sai Ting Chu, Yuen Kiu So, Albert Kar Kin Chung, Krystal Chi Kei Lee, Calvin Pak Wing Cheng, Chi Wing Law, Wai Chi Chan, Wing Chung Chang

**Affiliations:** ^1^Department of Psychiatry, School of Clinical Medicine, LKS Faculty of Medicine, The University of Hong Kong, Pokfulam, Hong Kong SAR, China; ^2^Department of Psychiatry, Queen Mary Hospital, Pokfulam, Hong Kong SAR, China; ^3^School of Public Health, LKS Faculty of Medicine, The University of Hong Kong, Pokfulam, Hong Kong SAR, China; ^4^State Key Laboratory of Brain and Cognitive Sciences, The University of Hong Kong, Pokfulam, Hong Kong SAR, China

**Keywords:** self-reported cognitive complaints, mental disorders, COVID-19, depression, traumatic stress symptoms, coping styles

## Abstract

**Introduction:**

The extent of cognitive impairment and its association with psychological distress among people with pre-existing mental illness during COVID-19 is understudied. This study aimed to investigate prevalence and correlates of subjective cognitive impairment (SCI) in Chinese psychiatric patients during fifth-wave of COVID-19 in Hong Kong (HK).

**Methods:**

Four-hundred-eight psychiatric outpatients aged 18–64 years were assessed with questionnaires between 28 March and 8 April 2022, encompassing illness profile, psychopathological symptoms, coping-styles, resilience, and COVID-19 related factors. Participants were categorized into moderate-to-severe and intact/mild cognitive impairment (CI+ vs. CI-) groups based on severity of self-reported cognitive complaints. Univariate and multivariate regression analyses were conducted to determine variables associated with CI+ status.

**Results:**

One-hundred-ninety-nine participants (48.8%) experienced CI+. A multivariate model on psychopathological symptoms found that depressive and post-traumatic-stress-disorder (PTSD)-like symptoms were related to CI+, while a multivariate model on coping, resilience and COVID-19 related factors revealed that avoidant coping, low resilience and more stressors were associated with CI+. Final combined model demonstrated the best model performance and showed that more severe depressive and PTSD-like symptoms, and adoption of avoidant coping were significantly associated with CI+.

**Conclusion:**

Almost half of the sample of psychiatric patients reported cognitive complaints during fifth-wave of COVID-19 in HK. Greater depressive and PTSD-like symptom severity, and maladaptive (avoidant) coping were found as correlates of SCI. COVID-19 related factors were not independently associated with SCI in psychiatric patients. Early detection with targeted psychological interventions may therefore reduce psychological distress, and hence self-perceived cognitive difficulties in this vulnerable population.

## 1. Introduction

Coronavirus disease (COVID-19) is an infectious disease caused by a new strain of severe acute respiratory syndrome coronavirus, SARS-CoV-2 (1). It first emerged in December 2019 in China and was declared as a global pandemic in March 2020 (2). COVID-19 brings about both physical and mental health complications. Adverse mental health outcomes such as increased stress, higher levels of worry and fatigue, pandemic-related fear and anxiety, depressive, anxiety and post-traumatic stress symptoms have been consistently reported in COVID-19 studies on the general population across different countries (3–7). The indirect effects of COVID-19 such as economic ramification (8) and social isolation (9, 10) due to public health policies further compromise individuals’ psychological well-being. In particular, individuals with pre-existing mental illness constitute one of the vulnerable populations in the pandemic. Literature showed that psychiatric patients had higher rates of COVID-19 infection (11, 12) and mortality (13, 14), and experienced greater psychological distress with more severe depressive and anxiety symptoms, and sleep disturbances (15, 16).

Owing to its high contagious nature, many countries have implemented a myriad of preventive measures in containing COVID-19 infection. In Hong Kong (HK), local infections waxed and waned in an early pandemic stage until an emergence of the Omicron variant in the community in late December 2021. Subsequently, a new wave of outbreak began (Supplementary Figure S1) and the 7-day rolling average of COVID-related deaths reached 3.73 per 1000 people at the peak, the highest worldwide, since Omicron variant was detected (17, 18). It was even estimated that approximately half of the HK population (i.e., 3.6 million) have contracted COVID-19 during the fifth wave by mid-March (19). Epidemiological control measures were further tightened, together with the consideration of implementing compulsory mass COVID-19 testing (and the associated lockdown measures) to the entire population by the government (20). Previous studies reported increased prevalence of depression and anxiety in the general population (21–23), and worsening of mood symptoms in psychiatric patients (24) during the early stage of COVID-19 in HK.

Of note, recent data have found that COVID-19 outbreak and its related psychological distress may be associated with poorer cognitive functioning in the general population (25, 26) and psychiatric patients (27). In particular, psychiatric patients may have already experienced certain extent of cognitive deficits due to their pre-existing mental illness, and may be therefore more susceptible to further cognitive deterioration in the midst of pandemic. Despite the significance of cognitive impairment on psychosocial functioning, there is a paucity of research specifically investigating cognitive complaints in psychiatric patients and its relationship with COVID-19 related factors. To this end, the current study aimed to: firstly, examine the prevalence of subjective cognitive impairment (SCI) in a representative cohort of Chinese patients with common and severe mental disorders during the fifth wave of COVID-19 pandemic in HK; and secondly, to identify correlates of SCI among psychiatric patients by comprehensively evaluating a wide array of factors encompassing socio-demographics, psychopathological symptoms, level of resilience, stress coping strategies and COVID-19 related variables.

## 2. Materials and methods

### 2.1. Participants and study setting

Participants aged 18–64 years were recruited from adult public psychiatric outpatient clinics between March 28 and April 8, 2022 in Hong Kong West Cluster, a catchment area with a population of approximately 550, 000. Participants who have learning disabilities, head injuries and neurological disease, and who could not understand written Chinese language were excluded. Participants’ principal ICD10 psychiatric diagnosis was ascertained by reviewing electronic medical records of psychiatric services (ICD10 classification is used for psychiatric diagnostic assignment in HK public healthcare system). Participants were further categorized into patients with common mental disorders (CMD, including depression and anxiety disorders) and patients with severe mental disorders (SMD, including schizophrenia-spectrum disorders, bipolar disorder and other non-affective psychoses). The study was approved by the Institutional Review Board of the University of Hong Kong / Hospital Authority Hong Kong West Cluster and all participants provided written informed consent.

### 2.2. Study assessment

The self-rated study assessment comprised four sections including socio-demographics and illness profile, psychopathological symptom severity, coping strategies and resilience, and COVID-19 related factors. It took approximately 15–20 min to complete the questionnaire. Illness profile included psychiatric diagnosis, comorbid substance/alcohol use disorder, history of psychiatric admission and length of receiving psychiatric service. Concerning psychopathological symptoms, depressive and anxiety symptom severity were assessed by Patient Health Questionnaire-9 (PHQ-9) (28, 29) and Generalised Anxiety Disorder-7 scale (GAD-7) (30, 31), respectively, with both scales using a 4-point Likert scale ranging from 0 (never) to 3 (nearly every day). A modified version of Impact of Event Scale–Revised (IES-R) (32, 33) specific to COVID-19 was administered to measure post-traumatic stress disorder (PTSD)-like symptoms in a 5-point Likert scale (0 [never] to 4 [always]). Sleep quality and disturbance was assessed using Insomnia Severity Index (ISI) (34, 35). Positive symptom subdomain items (4 items) of 15-item Community Assessment of Psychic Experiences Scale–Chinese version (CAPE-C15) (36) was employed to assess positive psychotic symptoms. Participants rated their frequency of positive symptoms on a 4-point Likert scale (1 [never] to 4 [nearly always]). We did not apply negative symptom subdomain items of CAPE-C15 to measure negative symptoms as previous studies suggested considerable overlap with depressive symptoms (27, 37). For all of the symptom scales, higher scores indicated greater symptom severity. Self-harm behavior during the fifth wave of COVID-19 was assessed. Subjective cognitive impairment (SCI) of participants was measured by a 5-item self-report questionnaire, adapted from Cognitive Complaints in Bipolar Disorder Rating Assessment (COBRA) (38, 39), which has been applied in a recent study examining SCI in psychiatric patients of CMD and SMD during COVID-19 lockdown (27). The adapted SCI questionnaire comprised 5 items that reflected cognitive complaints manifested in everyday scenario, including cognitive domains of attention, processing speed, memory, learning and executive function. Each item was rated on the frequency of self-reported cognitive complaints on a 4-point Likert scale, ranging from 0 (never) to 3 (nearly every day) (27).

Participants’ coping strategies were assessed by an adapted Coping Orientation to Problems Experienced Inventory–Brief (Brief-COPE) (40, 41), which used a 4-point Likert scale ranging from 0 (never) to 3 (always). The 14 items of the adapted Brief-COPE were grouped into 3 copying styles based on previous factor-analytic study (42), namely avoidant, emotion-focused and problem-focused coping styles for subsequent analysis. A higher item sum score indicated higher level of engagement in that particular coping style. The Brief Resilience Scale (BRS) (43, 44) was used to assess resilience levels on a 5-point Likert scale (1 [strongly disagree] to 5 [strongly agree]), with higher scores indicating greater resilience. Evaluation of COVID-19 related factors comprised items assessing history of contracting COVID-19 infection, receipt of vaccination, fear of contagion, time spent on reading COVID-19 related information, COVID-19 related stressors experienced, specific infection control measures (e.g., under quarantine, mandatory COVID-19 testing) experienced and associated distress. Details of assessment for COVID-19 related factors are summarized in Supplementary Table S1.

### 2.3. Statistical analysis

The study sample was subdivided into psychiatric patients with moderate-to-severe cognitive impairment (CI+ group) and intact or mild cognitive impairment (CI- group), based on the SCI questionnaire ratings. Following the method of a previous study examining SCI in psychiatric patients during COVID-19 (27), participants attaining a score ≥ 2 in one or more of the 5 items on the adapted SCI questionnaire were categorized as fulfilling CI+ status. A series of univariate binary logistic regression analyses were conducted to examine the association of CI+ status with socio-demographics, illness profile variables, psychopathological symptoms, coping styles, resilience, and COVID-19 related factors. Then, three sets of multivariate binary logistic regression analyses using forward Wald stepwise method were performed. The first multivariate model included psychopathological variables that were significantly related to CI+ status in the preceding univariate analyses to determine which symptom domains were independently associated with CI+ status. The second model included variables of coping style and resilience as well as COVID-19 related factors that were significantly related to CI+ status in prior univariate analyses. The final combined model incorporated variables that were statistically significant in the first and second models, as well as socio-demographic and illness-profile variables that were significantly related to CI+ status in univariate analyses. This final model would determine an array of correlates that were independently associated with CI+ status. Model performance was assessed using receiver-operating characteristic (ROC) curve, area under ROC curve (AUC) and McFadden pseudo-R^2^. An AUC value of 0.7 to 0.8 is regarded as acceptable, and a value of 0.8 to 0.9 is considered excellent (45). All missing data were imputed for five times using Multiple Imputation by Chained Equations (MICE) (46). All results reported were pooled according to Rubin’s rules (47). All analyses were conducted using R4.2.1, with significance level set as *p* < 0.05.

## 3. Results

### 3.1. Characteristics of the sample

A total of 415 participants were recruited. Seven participants were excluded due to missing data in the SCI questionnaire, resulting in 408 participants as the study sample for analysis. Of the 408 participants, 242 (59.3%) and 166 (40.7%) were CMD and SMD patients, respectively. The median duration of receiving public psychiatric care was 7.4 years. Based on the self-reported SCI assessment, 199 (48.8%) patients were categorized as CI+, while 209 patients (51.2%) were categorized as CI-. Characteristics of CI+ and CI- groups are summarized in Table 1.

**Table 1:** Descriptive and univariate logistic regression results of candidate variables for cognitive impairment status.

	CI- group (*n*=209)	CI+ group (*n*=199)	OR (95% CI)	*P*
Socio-demographics (*n*, %)
Gender (female)	126 (60.3%)	131 (65.8%)	1.25 (0.83-1.88)	0.283
Age, years
18-29	43 (20.6%)	47 (23.6%)	1.00 (Reference)	-
30-44	77 (36.8%)	70 (35.2%)	0.80 (0.48-1.35)	0.410
45-59	66 (31.2%)	62 (31.2%)	0.84 (0.49-1.42)	0.508
60 or above	14 (6.7%)	10 (5.0%)	0.64 (0.26-1.58)	0.336
Years of education
Secondary level or below	115 (55.0%)	116 (58.3%)	1.13 (0.76-1.68)	0.552
Tertiary level of above	90 (43.1%)	79 (39.7%)	1.00 (Reference)	-
Marital status
Single	123 (58.9%)	93 (46.7%)	1.00 (Reference)	-
Married	63 (30.1%)	67 (33.7%)	1.38 (0.89-2.15)	0.154
Divorced/ widowed	18 (8.6%)	36 (18.1%)	2.58 (1.37-4.83)	0.003
Employment status
Employed	160 (76.6%)	135 (67.8%)	1.00 (Reference)	-
Unemployed	36 (17.2%)	53 (26.6%)	1.74 (1.08-2.83)	0.024
Retired	11 (5.3%)	7 (3.5%)	0.79 (0.29-2.13)	0.635
Housing area^a^ (square feet)				
Less than 300	48 (23.0%)	74 (37.2%)	1.90 (1.21-2.97)	0.005
301-800	132 (63.2%)	106 (53.3%)	1.00 (Reference)	-
Greater than 800	22 (10.5%)	14 (7.0%)	0.77 (0.38-1.58)	0.473
Living alone (yes)	26 (12.4%)	38 (19.1%)	0.64 (0.37-1.09)	0.100
Monthly household income (HKD)				
25000 or below	128 (61.2%)	135 (67.8%)	1.46 (0.95-2.22)	0.081
Above 25000^b^	73 (34.9%)	54 (27.1%)	1.00 (Reference)	-
Chronic physical comorbidity (yes)	60 (28.7%)	54 (27.1%)	0.84 (0.59-1.20)	0.331
Illness profile (*n*, %)
Psychiatric diagnosis
Common mental disorders	109 (52.2%)	133 (66.8%)	1.00 (Reference)	-
Severe mental disorders	100 (47.8%)	66 (33.2%)	0.54 (0.36-0.81)	0.003
Alcohol/ Substance use disorder	6 (2.9%)	22 (11.1%)	4.25 (1.68-10.7)	0.002
History of psychiatric hospitalization	96 (45.9%)	76 (38.2%)	0.74 (0.49-1.11)	0.147
Years in psychiatric service (mean, SD)	8.6 (6.3)	7.8 (6.2)	0.98 (0.94-1.01)	0.146
Psychopathological symptoms (mean, SD)
Depressive symptoms (PHQ-9)	5.3 (5.1)	14.1 (6.6)	1.26 (1.21-1.32)	<0.001
Anxiety symptoms (GAD-7)	4.1 (4.7)	11.5 (6.2)	1.25 (1.19-1.31)	<0.001
PTSD-like symptoms (IES-R)	4.4 (4.6)	10.3 (5.9)	1.23 (1.17-1.29)	<0.001
Insomnia symptoms (ISI)	8.2 (6.2)	15.4 (6.7)	1.18 (1.13-1.22)	<0.001
Positive psychotic symptoms (CAPE-C15)	4.8 (1.4)	6.5 (2.5)	1.55 (1.36-1.76)	<0.001
Self-harm behaviour (*n*, %)	7 (3.4%)	34 (17.1%)	5.95 (2.56-13.8)	<0.001
Coping and resilience (mean, SD)
Avoidant coping	3.8 (2.5)	6.1 (2.4)	1.45 (1.31-1.60)	<0.001
Emotion-focused coping	6.3 (3.6)	7.4 (2.9)	1.09 (1.03-1.17)	0.004
Problem-focused coping	3.7 (2.3)	4.0 (1.8)	1.07 (0.98-1.18)	0.141
Resilience	19.1 (4.0)	15.3 (4.3)	0.80 (0.75-0.85)	<0.001
COVID-19 related factors (*n*, %)
Contracting COVID-19 infection in 5^th^ wave	54 (25.8%)	46 (23.1%)	0.88 (0.56-1.39)	0.591
COVID-19 vaccine doses received (≥2)	183 (87.6%)	159 (79.9%)	0.52 (0.28-0.96)	0.036
Number of COVID-19 stressors			1.38 (1.27-1.50)	<0.001
0-2	132 (63.2%)	48 (24.1%)	-	-
3-5	44 (21.1%)	58 (29.1%)	-	-
6-8	32 (15.3%)	89 (44.7%)	-	-
Fear of contagion (mean, SD)	3.7 (2.8)	5.4 (3.3)	1.19 (1.11-1.28)	<0.001
Time spent on reading COVID-19 related information				
None	19 (9.1%)	11 (37.9%)	1.00 (Reference)	-
1-3 hours	168 (80.4%)	145 (69.4%)	1.49 (0.68-3.27)	0.320
>3 hours	18 (8.6%)	37 (18.6%)	3.56 (1.40-9.05)	0.008
Mandatory testing or quarantine	35 (16.7%)	35 (17.6%)	1.03 (0.62-1.73)	0.897
Distress by social-distancing measures (mean, SD)	4.2 (3.1)	5.9 (3.3)	1.17 (1.10-1.25)	<0.001

*CI-,* None to mild cognitive impairment; *CI,+* Moderate to severe cognitive impairment, *CI,* confidence interval; *CAPE-C15,* Community Assessment of Psychic Experiences Scale – Chinese version (15 items); *COVID-19,* Coronavirus disease 2019; *GAD-7,* Generalised Anxiety Disorder scale; *HKD,* Hong Kong dollars; *IES-R,* Impact of Event Scale – Revised; *ISI,* Insomnia Severity Index; *OR,* Odds ratio; *PHQ-9,* Patient Health Questionnaire; *PTSD,* Post-traumatic stress disorder; *SD,* Standard deviations.

a The median of housing area excluding common area in Hong Kong is approximately 430 square feet according to the Population Census 2021.

b The median of monthly household income is HKD27,100 according to the Hong Kong Census and Statistics Department April-June, 2022. As of 9 Nov 2022,

1 HKD = 0.13 USD.

### 3.2. Univariate regression analyses for subjective cognitive impairment

Results of univariate regression analyses examining the associations of CI+ status with variables encompassing socio-demographics, illness profiles, psychopathological symptoms, coping styles and resilience, and COVID-19 related factors are shown in Table 1. Marital status (divorced / widowed), unemployment, median housing area < 300 sq. ft., CMD diagnosis, and substance/alcohol use disorder were associated with increased likelihood of CI+. Patients in CI+ group had higher scores in PHQ-9, GAD-7, IES-R, ISI and CAPE-15C positive symptom subdomain, and were more likely to commit self-harm behavior during the fifth wave of COVID-19 than those in CI- group. Furthermore, CI+ group was more likely to adopt avoidant and emotion-focused coping styles and had lower BRS score relative to CI- group. Regarding COVID-19 related factors, CI+ group experienced greater number of stressors and greater fear of contracting COVID-19, was more likely to have received <2 COVID-19 vaccine doses and spent >3 h reading COVID-19 information, and was more distressed by social-distancing measures than CI- group.

### 3.3. Multivariate and final combined regression models for subjective cognitive impairment

**Figure 1:**
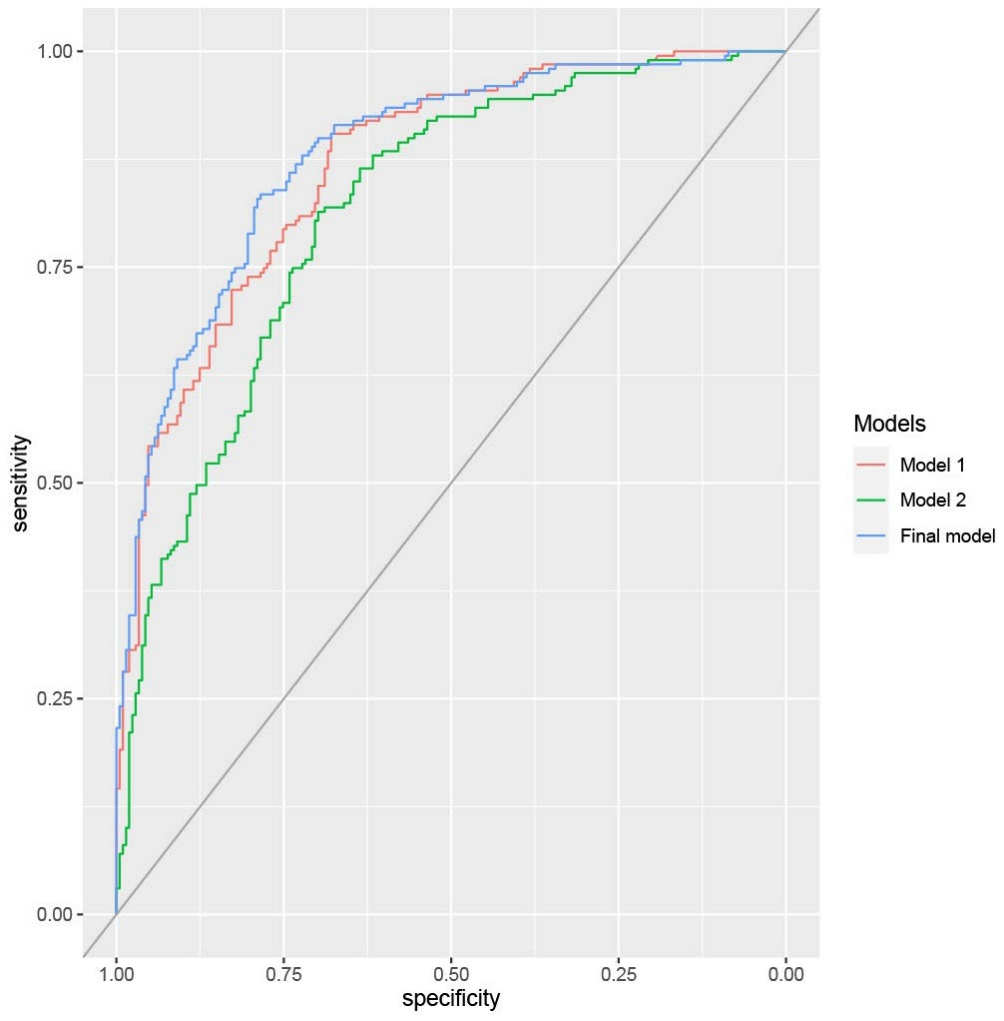
Receiver Operating Characteristic (ROC) curves of the multivariate logistic regression models for prediction of cognitive impairment status. Model 1: Prediction of psychopathological symptoms on cognitive impairment. Model 2: Prediction of resilience, coping and COVID-19 related factors on cognitive impairment. Final model: Combined factors of models 1 and 2 for predicting cognitive impairment.

As shown in Table 2, multivariate regression model 1 on psychopathological symptoms revealed that more severe depressive and PTSD-like symptoms were significantly associated with CI+. Model 2 showed that avoidant coping style, lower resilience and greater number of COVID-19 related stressors were significantly associated with CI+ status (Table 2). Final combined model found that depressive symptoms, PTSD-like symptoms and avoidant coping style were independently associated with CI+ (Table 3). The final model demonstrated superior model performance to models 1 and 2 in determining CI+ status as evidenced by having the highest AUC value and explained variance (Figure 1; Tables 2, 3).

**Table 2:** Multivariate logistic regression analyses on psychopathological symptoms and coping, resilience & COVID-19 related factors for cognitive impairment status.

Model 1: Psychopathological symptoms				
Variables	OR (95% CI)	*P*	AUC	McFadden pseudo R^2^
Depressive symptoms (PHQ-9)	1.16 (1.08-1.25)	<0.001		
Anxiety symptoms (GAD-7)	1.00 (0.92-1.09)	0.951		
PTSD-like symptoms (IES-R)	1.08 (1.02-1.16)	0.012		
Insomnia symptoms (ISI)	1.04 (0.99-1.09)	0.138		
Psychotic symptoms (CAPE-C15)	1.10 (0.95-1.28)	0.198		
Self-harm behaviour	1.55 (0.57-4.21)	0.389		
	-	-	0.866	0.336
Model 2: Coping, resilience and COVID-19 related factors				
Avoidant coping	1.43 (1.25-1.63)	<0.001		
Emotion-focused coping	0.92 (0.82-1.03)	0.161		
Problem-focused coping	0.96 (0.80-1.15)	0.644		
Resilience	0.86 (0.80-0.92)	<0.001		
Contracting COVID-19 in fifth wave	0.83 (0.47-1.50)	0.521		
COVID-19 vaccine doses received (≥2)	0.76 (0.36-1.62)	0.474		
Number of COVID-19 stressors	1.15 (1.03-1.29)	0.011		
Fear of contagion	1.07 (0.97-1.17)	0.171		
Time spent on reading COVID-19 related information				
None	1.00 (Reference)			
1-3 hours	1.10 (0.39-3.16)	0.852		
>3 hours	1.77 (0.51-6.16)	0.368		
Distress by social-distancing measures	1.00 (0.92-1.09)	0.946		
	-	-	0.840	0.272

*AUC,* Area under the receiver-operating characteristic (ROC) curve; *CAPE-C15,* Community.

Assessment of Psychic Experiences Scale – Chinese version (15 items); *CI,* Confidence interval; *COVID-19,* Coronavirus disease 2019; *GAD-7,* Generalised Anxiety Disorder scale; *IES-R,* Impact of Event Scale – Revised; *ISI,* Insomnia Severity Index; *OR,* Odds ratio; *PHQ-9,* Patient Health Questionnaire; *PTSD,* Post-traumatic stress disorder.

**Table 3:** Final multivariate logistic regression model on cognitive impairment status.

Variables	OR (95% CI)	*P*	AUC	McFadden pseudo-R^2^
Marital status				
Single	1.00 (Reference)			
Married	1.26 (0.68-2.34)	0.464		
Divorced/ widowed	0.94 (0.45-1.94)	0.893		
Employment status				
Employed	1.00 (Reference)			
Unemployed	0.94 (0.45-1.94)	0.861		
Retired	1.89 (0.51-7.05)	0.343		
Housing area (square feet)				
Less than 300	1.80 (0.94-3.42)	0.074		
301-800	1.00 (Reference)			
Greater than 800	0.59 (0.22-1.60)	0.303		
Psychiatric diagnosis				
Common mental disorders	1.00 (Reference)			
Severe mental disorders	1.73 (0.93-3.23)	0.084		
Alcohol/ Substance use disorder	1.67 (0.48-5.80)	0.420		
Depressive symptoms (GAD-7)	1.19 (1.11-1.26)	<0.001		
PTSD-like symptoms (IES-R)	1.07 (1.00-1.14)	0.037		
Avoidant coping	1.21 (1.07-1.38)	0.003		
Resilience	0.94 (0.87-1.02)	0.129		
Number of COVID-related stressors	1.03 (0.91-1.16)	0.626		
	-	-	0.880	0.370

*AUC,* Area under curve; *CI,* Confidence interval; *GAD-7,* Generalised Anxiety Disorder scale; *IES-R,* Impact of Event Scale – Revised; *OR,* Odds ratio; *PHQ-9,* Patient Health Questionnaire; *PTSD,* Post-traumatic stress disorder.

## 4. Discussion

The current study aimed to examine the prevalence and correlates of SCI in Chinese psychiatric patients during the fifth wave of COVID-19 in HK. To our knowledge, this is one of the few studies to investigate SCI in psychiatric patients in relation to COVID-19 pandemic, and is the first of its kind in Asia and in Chinese population. Our results showed that almost half of our patient sample reported moderate-to-severe SCI. This is slightly higher than that observed in a recent Spanish study which found that 40.9% of psychiatric patients reported cognitive complaints during COVID-19 lockdown (27).

Our results from a multivariate model 1 on psychopathological symptoms revealed that depressive and PTSD-like symptoms were related to CI+ status. Importantly, these two symptom domains remained significant in the final combined model, indicating that more severe depressive and PTSD-like symptoms were independently associated with moderate-to-severe SCI. In fact, the observed association between depression and CI+ is consistent with a prior Spanish study which demonstrated that psychiatric patients with greater depressive symptom severity were significantly more likely to be categorized in CI+ group (27). It is noteworthy that this Spanish study also found a significant association between CI+ and negative symptoms (as measured by CAPE), which, nonetheless, are greatly overlapped with (and thus likely represent) depressive symptoms (27). There is also ample evidence supporting the link between depression and cognitive impairment. Literature has shown that patients with depression exhibit cognitive dysfunction during an acute phase as well as in remission (48–50). Alternatively, the significant relationship between PTSD-like symptoms and CI+ status corroborates with previous findings suggesting that the pandemic can act as a traumatic stressor, which may compromise individuals’ cognitive functions *via* the process of maladaptive mind wandering (51). Briefly, maladaptive mind wandering has been put forward as an important pathway mediating the association between PTSD-like symptoms and cognitive impairment by competing for the limited cognitive resources such as working memory capacity (52). In the context of COVID-19, maladaptive mind wandering comprises persistent worries of contagion and other pandemic-related adverse consequences, increased alertness and self-monitoring of physical symptoms, and constant checking of COVID-19 related news, to name a few (51). Taken together, our findings on psychopathological symptoms indicate that early recognition and prompt intervention (e.g., psychological treatments) of depressive and PTSD-like symptoms emerged in the pandemic not only alleviate symptom severity but may also reduce the risk of developing SCI in psychiatric patients.

We have conducted a comprehensive evaluation of coping strategies, resilience and COVID-19 related factors among psychiatric patients. Our multivariate model 2 showed that adoption of avoidant coping style, lower resilience and a greater number of stressors were related to CI+ status. The final model incorporating psychopathological symptoms as well as socio-demographics and illness profiles indicated that avoidant coping style remained significantly associated with CI+. It is acknowledged that avoidant coping is characterized by making cognitive and behavioral efforts in evading traumatic-related stressors (53). An extensive literature has documented the bi-directional association between avoidant coping strategies and various psychopathological symptoms including PTSD symptoms (54, 55), depression and anxiety (56). Recent data have also found that individuals with more frequent use of avoidant coping strategies experienced more depressive and anxiety symptoms during COVID-19 lockdown (57). Given its close relationship with PTSD-like, depressive and anxiety symptoms, avoidant coping style may thus exert an indirect effect on the development of SCI *via* manifestations of these psychopathological symptoms. Conversely, an earlier Spanish study demonstrated that positive coping strategies including performing physical and relaxing activities, and maintaining a routine during the lockdown were protective factors against cognitive complaints during lockdown, albeit these coping variables became non-significant in their final combined model incorporating psychopathological symptoms (27). Of note, we failed to identify any COVID-19 related factors that were independently associated with CI+. It is possible that the potential effect of COVID-19 related factors on SCI could be fully mediated by psychopathological symptoms and maladaptive coping styles. Owing to the paucity of existing data, further research is required to verify our findings on the relationship between pandemic-related factors and SCI in psychiatric patients.

This study has several methodological limitations. First, although the SCI questionnaire was adapted from COBRA, an established self-report measure of cognitive complaints in psychiatric patients, and has been adopted in a recent study examining SCI in patients with CMD and SMD during pandemic, further research is still needed to verify its psychometric properties. Second, our results were based on patients’ self-perceived cognitive complaints rather than objective cognitive assessment, which was not included in the study. This precludes us from clarifying whether our findings on SCI would be discrepant from data obtained *via* objective measures. Notably, there is evidence indicating poor concordance between subjective cognitive ratings and objective cognitive performance in healthy participants (58, 59) and psychiatric samples (60, 61). It is postulated that SCI and objectively-measured cognitive functions may represent two relatively distinct, albeit related, constructs. An inherent difference in settings between objective cognitive evaluation using standardized battery conducted in laboratory environment and self-perceived deficits emerged in unstructured real-world situations may also contribute to a lack of concurrence between these two cognitive measures. Third, the cross-sectional study design precludes establishment of causal relationship. Longitudinal research is warranted to determine the course of SCI over time and its predictors. Fourth, we did not collect data regarding the use of pharmacological or psychological treatment, and side-effects of psychotropic medications. Thus, we were not able to assess the potential effect of these treatment modalities on mental health outcomes and cognitive impairment. Lastly, as HK is a highly urbanized city and is categorized by the World Bank as a high-income economy (62), our findings may not be generalizable to mainland China or other Asian regions. Differences in ethno-cultural backgrounds as well as access to and quality of healthcare services across regions may further limit the generalizability of our results to other countries.

In conclusion, our study shows that moderate-to-severe cognitive complaints are prevalent in Chinese psychiatric outpatients during the fifth wave of COVID-19 pandemic. Depressive symptoms, PTSD-like symptoms and avoidant coping style are found to be correlates of CI+ status. Our results have several clinical implications. Although COVID-19 related factors are not independently associated with subjective cognitive impairment, the susceptibility of patients with pre-existing mental illness to COVID-19 related stressors and their ramifications on exacerbation of psychopathological symptoms and the overall psychological wellbeing should not be overlooked. Given the increased vulnerability to exacerbation of pre-existing cognitive dysfunction among psychiatric patients amidst the pandemic (and future public health crisis), early detection and psychosocial interventions of psychopathological symptoms, in particular depressive and PTSD-like symptoms, as well as rectification of maladaptive coping strategies are hence, crucial to minimize negative mental health impact and cognitive impairment. Exploration and development of effective strategies to promote personal resilience and the use of positive coping methods may also enhance protection against elevated risk of cognitive complaints. Prospective follow-up investigation is required to track the longitudinal trajectories of SCI and its predictors in relation to the subsequent course of pandemic and the post-pandemic era as well as the accompanied changes in public health policy measures.

## Data availability statement

The raw data supporting the conclusions of this article will be made available by the authors, without undue reservation.

## Ethics statement

The studies involving humans were approved by Institutional Review Board of the University of Hong Kong/Hospital Authority Hong Kong West Cluster. The studies were conducted in accordance with the local legislation and institutional requirements. The participants provided their written informed consent to participate in this study.

## Author contributions

Wic, EC, and CW designed and conceptualized the study. VF, JaC, JoC, and RC conducted data collection. VF and JaC conducted statistical analysis. VF wrote the first draft of the manuscript. Wic, VF, EC, CW, and CL interpreted the study data. Wic and VF revised and finalized the manuscript. All authors contributed to the article and approved the submitted version.

## Funding

The study was supported by the Hong Kong Research Grants Council (grant number: 10617014). The funders had no role in study design, data collection, data analysis, interpretation of the data, manuscript preparation or journal submission.

## Conflict of interest

The authors declare that the research was conducted in the absence of any commercial or financial relationships that could be construed as a potential conflict of interest.

## Publisher’s note

All claims expressed in this article are solely those of the authors and do not necessarily represent those of their affiliated organizations, or those of the publisher, the editors and the reviewers. Any product that may be evaluated in this article, or claim that may be made by its manufacturer, is not guaranteed or endorsed by the publisher.

## References

[1] Wang C Horby PW Hayden FG Gao GF. A novel coronavirus outbreak of global health concern. Lancet. (2020) 395:470–3. doi: 10.1016/S0140-6736(20)30185-9, PMID: 31986257 PMC7135038

[2] World Health Organization (WHO). Timeline: WHO's COVID-19 response (2020). Available at: https://www.who.int/emergencies/diseases/novel-coronavirus-2019/interactive-timeline#! (Accessed December 18, 2022).

[3] Blasco-Belled A Tejada-Gallardo C Fatsini-Prats M Alsinet C. Mental health among the general population and healthcare workers during the COVID-19 pandemic: a meta-analysis of well-being and psychological distress prevalence. Curr Psychol. (2022):1–12. doi: 10.1007/s12144-022-02913-6, PMID: 35250245 PMC8887799

[4] Dragioti E Li H Tsitsas G Lee KH Choi J Kim J. A large-scale meta-analytic atlas of mental health problems prevalence during the COVID-19 early pandemic. J Med Virol. (2022) 94:1935–49. doi: 10.1002/jmv.27549, PMID: 34958144 PMC9015528

[5] Brailovskaia J Cosci F Mansueto G Margraf J. The relationship between social media use, stress symptoms and burden caused by coronavirus (Covid-19) in Germany and Italy: a cross-sectional and longitudinal investigation. J Affect Disord Rep. (2021) 3:100067. doi: 10.1016/j.jadr.2020.100067, PMID: 35434690 PMC8995101

[6] Mansueto G Lopes FL Grassi L Cosci F. Impact of COVID-19 outbreak on Italian healthcare workers versus general population: results from an online survey. Clin Psychol Psychother. (2021) 28:1334–45. doi: 10.1002/cpp.2644, PMID: 34255890 PMC8426916

[7] Alhakami A Salem V Alateeq D Nikčević AV Marci T Palmieri S. The Arab COVID-19 anxiety syndrome scale (C-19ASS): COVID-19 anxiety syndrome and psychological symptoms in the Saudi Arabian population. Clin Psychol Psychother. (2023). doi: 10.1002/cpp.2860, PMID: 37183315

[8] Ueda M Stickley A Sueki H Matsubayashi T. Mental health status of the general population in Japan during the COVID-19 pandemic. Psychiatry Clin Neurosci. (2020) 74:505–6. doi: 10.1111/pcn.13105, PMID: 32609413 PMC7361838

[9] Cavicchioli M Ferrucci R Guidetti M Canevini MP Pravettoni G Galli F. What will be the impact of the Covid-19 quarantine on psychological distress? Considerations based on a systematic review of pandemic outbreaks. Healthcare. (2021) 9:101. doi: 10.3390/healthcare9010101, PMID: 33477981 PMC7835976

[10] Galea S Merchant RM Lurie N Galea S. The mental health consequences of COVID-19 and physical distancing: the need for prevention and early intervention. JAMA Intern Med. (2020) 180:817–8. doi: 10.1001/jamainternmed.2020.1562 32275292

[11] Taquet M Luciano S Geddes JR Harrison PJ. Bidirectional associations between COVID-19 and psychiatric disorder: retrospective cohort studies of 62 354 COVID-19 cases in the USA. Lancet Psychiatry. (2021) 8:130–40. doi: 10.1016/S2215-0366(20)30462-4, PMID: 33181098 PMC7820108

[12] Wang Q Xu R Volkow ND. Increased risk of COVID-19 infection and mortality in people with mental disorders: analysis from electronic health records in the United States. World Psychiatry. (2021) 20:124–30. doi: 10.1002/wps.20806, PMID: 33026219 PMC7675495

[13] Fond G Nemani K Etchecopar-Etchart D Loundou A Goff DC Lee SW. Association between mental health disorders and mortality among patients with COVID-19 in 7 countries: a systematic review and meta-analysis. JAMA Psychiat. (2021) 78:1208–17. doi: 10.1001/jamapsychiatry.2021.2274, PMID: 34313711 PMC8317055

[14] Toubasi AA AbuAnzeh RB Tawileh HB Aldebei RH Alryalat SA. A meta-analysis: the mortality and severity of COVID-19 among patients with mental disorders. Psychiatry Res. (2021) 299:113856. doi: 10.1016/j.psychres.2021.113856, PMID: 33740483 PMC7927594

[15] Iasevoli F Fornaro M D'Urso G Galletta D Casella C Paternoster M. COVID-19 in psychiatry study group. Psychological distress in patients with serious mental illness during the COVID-19 outbreak and one-month mass quarantine in Italy. Psychol Med. (2021) 51:1054–6. doi: 10.1017/S0033291720001841, PMID: 32423496 PMC7261960

[16] Solé B Verdolini N Amoretti S Montejo L Rosa AR Hogg B. Effects of the COVID-19 pandemic and lockdown in Spain: comparison between community controls and patients with a psychiatric disorder. Preliminary results from the BRIS-MHC study. J Affect Disord. (2021) 281:13–23. doi: 10.1016/j.jad.2020.11.099, PMID: 33279864 PMC7683299

[17] Cheung PH Chan CP Jin DY. Lessons learned from the fifth wave of COVID-19 in Hong Kong in early 2022. Emerg Microbes Infect. (2022) 11:1072–8. doi: 10.1080/22221751.2022.2060137, PMID: 35348429 PMC9004509

[18] Lee RS Hermens DF Porter MA Redoblado-Hodge MA. A meta-analysis of cognitive deficits in first-episode major depressive disorder. J Affect Disord. (2012) 140:113–24. doi: 10.1016/j.jad.2011.10.023, PMID: 22088608

[19] Modelling the fifth wave of COVID-19 in Hong Kong (2022). Available at: https://www.med.hku.hk/en/news/press/media/DF5A2F6918764DC4B6517CE7B5F2796B.ashx (Accessed November 24, 2022).

[20] Chau C. Explainer: how Hong Kong’s Covid policies shifted during the fifth wave crisis (2022). Available at: https://hongkongfp.com/2022/03/20/explainer-how-govt-covid-19-policies-have-shifted-since-the-beginning-of-the-fifth-wave/ (Accessed December 18, 2022).

[21] Choi EP Hui BP Wan EY. Depression and anxiety in Hong Kong during COVID-19. Int J Environ Res Public Health. (2020) 17:3740. doi: 10.3390/ijerph17103740, PMID: 32466251 PMC7277420

[22] Tso IF Park S. Alarming levels of psychiatric symptoms and the role of loneliness during the COVID-19 epidemic: a case study of Hong Kong. Psychiatry Res. (2020) 293:113423. doi: 10.1016/j.psychres.2020.113423, PMID: 32871487 PMC7443338

[23] Zhao SZ Wong JY Luk TT Wai AK Lam TH Wang MP. Mental health crisis under COVID-19 pandemic in Hong Kong. China Int J Infect Dis. (2020) 100:431–3. doi: 10.1016/j.ijid.2020.09.030, PMID: 32947051 PMC7492140

[24] Li JT Lee CP Tang WK. Changes in mental health among psychiatric patients during the COVID-19 pandemic in Hong Kong—a cross-sectional study. Int J Environ Res Public Health. (2022) 19:1181. doi: 10.3390/ijerph19031181, PMID: 35162205 PMC8834986

[25] Fiorenzato E Zabberoni S Costa A Cona G. Cognitive and mental health changes and their vulnerability factors related to COVID-19 lockdown in Italy. PLoS One. (2021) 16:e0246204. doi: 10.1371/journal.pone.0246204, PMID: 33503055 PMC7840042

[26] Kira IA Alpay EH Ayna YE Shuwiekh HA Ashby JS Turkeli A. The effects of COVID-19 continuous traumatic stressors on mental health and cognitive functioning: a case example from Turkey. Curr Psychol. (2021) 41:7371–82. doi: 10.1007/s12144-021-01743-2 33897228 PMC8057920

[27] Montejo L Solé B Verdolini N Martínez-Arán A del Mar BC Radua J. Self-reported neurocognitive symptoms during COVID-19 lockdown and its associated factors in a sample of psychiatric patients. Results from the BRIS-MHC study. Eur Neuropsychopharmacol. (2021) 53:7–18. doi: 10.1016/j.euroneuro.2021.07.006, PMID: 34348213 PMC8619656

[28] Kroenke K Spitzer RL Williams JB. The PHQ-9: validity of a brief depression severity measure. J Gen Intern Med. (2001) 16:606–13. doi: 10.1046/j.1525-1497.2001.016009606.x, PMID: 11556941 PMC1495268

[29] Yu X Tam WW Wong PT Lam TH Stewart SM. The patient health Questionnaire-9 for measuring depressive symptoms among the general population in Hong Kong. Compr Psychiatry. (2012) 53:95–102. doi: 10.1016/j.comppsych.2010.11.002, PMID: 21193179

[30] Spitzer RL Kroenke K Williams JB Löwe B. A brief measure for assessing generalized anxiety disorder: the GAD-7. Arch Intern Med. (2006) 166:1092–7. doi: 10.1001/archinte.166.10.1092 16717171

[31] Tong X An D McGonigal A Park SP Zhou D. Validation of the generalized anxiety Disorder-7 (GAD-7) among Chinese people with epilepsy. Epilepsy Res. (2016) 120:31–6. doi: 10.1016/j.eplepsyres.2015.11.019, PMID: 26709880

[32] Guo J Yang L Xu Y Zhang C Luo X Liu S. Prevalence and risk factors associated with insomnia symptoms among the Chinese general public after the coronavirus disease 2019 epidemic was initially controlled. Nat Sci Sleep. (2021) 13:703–12. doi: 10.2147/NSS.S307996, PMID: 34104023 PMC8180302

[33] Wu KK Chan KS. The development of the Chinese version of impact of event scale - revised (CIES-R). Soc Psychiatry Psychiatr Epidemiol. (2003) 38:94–8. doi: 10.1007/s00127-003-0611-x, PMID: 12563552

[34] Bastien CH Vallières A Morin CM. Validation of the insomnia severity index as an outcome measure for insomnia research. Sleep Med. (2001) 2:297–307. doi: 10.1016/S1389-9457(00)00065-4 11438246

[35] Yeung WF Chung KF Zhang SP Yap TG Law AC. Electroacupuncture for primary insomnia: a randomized controlled trial. Sleep. (2009) 32:1039–47. doi: 10.1093/sleep/32.8.1039, PMID: 19725255 PMC2717194

[36] Mark W Toulopoulou T. Validation of the Chinese version of community assessment of psychic experiences (CAPE) in an adolescent general population. Asian J Psychiatry. (2017) 26:58–65. doi: 10.1016/j.ajp.2017.01.012, PMID: 28483093

[37] Krynicki CR Upthegrove R Deakin JFW Barnes TRE. The relationship between negative symptoms and depression in schizophrenia: a systematic review. Acta Psychiatr Scand. (2018) 137:380–90. doi: 10.1111/acps.12873, PMID: 29532909

[38] Rosa AR Mercadé C Sánchez-Moreno J Solé B del Mar BC Torrent C. Validity and reliability of a rating scale on subjective cognitive deficits in bipolar disorder (COBRA). J Affect Disord. (2013) 150:29–36. doi: 10.1016/j.jad.2013.02.022, PMID: 23497792

[39] Xiao L Lin X Wang Q Lu D Tang S. Adaptation and validation of the “cognitive complaints in bipolar disorder rating assessment” (COBRA) in Chinese bipolar patients. J Affect Disord. (2015) 173:226–31. doi: 10.1016/j.jad.2014.11.011, PMID: 25462421

[40] Carver CS. You want to measure coping but your protocol is too long: consider the brief cope. Int J Behav Med. (1997) 4:92–100. doi: 10.1207/s15327558ijbm0401_6, PMID: 16250744

[41] Ye S. A longitudinal study of subjective well-being among Chinese university students: The roles of personality, attribution, and coping. [Doctoral Thesis]. Hong Kong: University of Hong Kong (2008).

[42] Dias C Cruz JF Fonseca AM. The relationship between multidimensional competitive anxiety, cognitive threat appraisal, and coping strategies: a multi-sport study. Int J Sport Exerc Psychol. (2012) 10:52–65. doi: 10.1080/1612197X.2012.645131

[43] Hsin YZ. Reliability and validity of the brief resilience scale. [Master thesis]. Taiwan: National Pingtung University (2020).

[44] Smith BW Dalen J Wiggins K Tooley E Christopher P Bernard J. The brief resilience scale: assessing the ability to bounce back. Int J Behav Med. (2008) 15:194–200. doi: 10.1080/10705500802222972, PMID: 18696313

[45] Hosmer DW Jr Lemeshow S Sturdivant RX. Applied logistic regression. Hoboken, New Jersey: John Wiley & Sons (2013).

[46] van Buuren S Groothuis-Oudshoorn K. Mice: multivariate imputation by chained equations in R. J Stat Softw. (2011) 45:1–67. doi: 10.18637/jss.v045.i03

[47] Rubin DB. Multiple imputation for nonresponse in surveys. New York: John Wiley & Sons (2004).

[48] Conradi HJ Ormel J de Jonge P. Presence of individual (residual) symptoms during depressive episodes and periods of remission: a 3-year prospective study. Psychol Med. (2010) 41:1165–74. doi: 10.1017/S0033291710001911 20932356

[49] Lee P. Covid-19 in data: 7 charts showing Hong Kong’s deadly omicron outbreak (2022). Available at: https://hongkongfp.com/2022/03/12/covid-19-in-data-6-charts-showing-hong-kongs-deadly-omicron-outbreak/ (Accessed December 18, 2022).

[50] Semkovska M Quinlivan L O'Grady T Johnson R Collins A O'Connor J. Cognitive function following a major depressive episode: a systematic review and meta-analysis. Lancet Psychiatry. (2019) 6:851–61. doi: 10.1016/S2215-0366(19)30291-3, PMID: 31422920

[51] Boals A Banks JB. Stress and cognitive functioning during a pandemic: thoughts from stress researchers. Psychol Trauma. (2020) 12:S255–7. doi: 10.1037/tra0000716, PMID: 32463284

[52] Banks JB Boals A. Understanding the role of mind wandering in stress-related working memory impairments. Cogn Emot. (2017) 31:1023–30. doi: 10.1080/02699931.2016.1179174, PMID: 27144890

[53] Penley JA Tomaka J Wiebe JS. The association of coping to physical and psychological health outcomes: a meta-analytic review. J Behav Med. (2002) 25:551–603. doi: 10.1023/A:1020641400589 12462958

[54] Thompson NJ Fiorillo D Rothbaum BO Ressler KJ Michopoulos V. Coping strategies as mediators in relation to resilience and posttraumatic stress disorder. J Affect Disord. (2018) 225:153–9. doi: 10.1016/j.jad.2017.08.049, PMID: 28837948 PMC5626644

[55] Tiet QQ Rosen C Cavella S Moos RH Finney JW Yesavage J. Coping, symptoms, and functioning outcomes of patients with posttraumatic stress disorder. J Trauma Stress. (2006) 19:799–811. doi: 10.1002/jts.20185 17195979

[56] Grant DM Wingate LR Rasmussen KA Davidson CL Slish ML Rhoades-Kerswill S. An examination of the reciprocal relationship between avoidance coping and symptoms of anxiety and depression. J Soc Clin Psychol. (2013) 32:878–96. doi: 10.1521/jscp.2013.32.8.878

[57] Fluharty M Bu F Steptoe A Fancourt D. Coping strategies and mental health trajectories during the first 21 weeks of COVID-19 lockdown in the United Kingdom. Soc Sci Med. (2021) 279:113958. doi: 10.1016/j.socscimed.2021.113958, PMID: 33965772 PMC9756769

[58] Chaytor N Schmitter-Edgecombe M. The ecological validity of neuropsychological tests: a review of the literature on everyday cognitive skills. Neuropsychol Rev. (2003) 13:181–97. doi: 10.1023/B:NERV.0000009483.91468.fb, PMID: 15000225

[59] van der Elst W van Boxtel MP van Breukelen GJ Jolles J. A large-scale cross-sectional and longitudinal study into the ecological validity of neuropsychological test measures in neurologically intact people. Arch Clin Neuropsychol. (2008) 23:787–800. doi: 10.1016/j.acn.2008.09.002, PMID: 18930628

[60] Chang WC Chan TC Chiu SS Hui CL Chan SK Lee EH. Self-perceived cognitive functioning and its relationship with objective performance in first-episode schizophrenia: the subjective cognitive impairment scale. Compr Psychiatry. (2015) 56:42–50. doi: 10.1016/j.comppsych.2014.10.004, PMID: 25459418

[61] Serra-Blasco M Torres IJ Vicent-Gil M Goldberg X Navarra-Ventura G Aguilar E. Discrepancy between objective and subjective cognition in major depressive disorder. Eur Neuropsychopharmacol. (2019) 29:46–56. doi: 10.1016/j.euroneuro.2018.11.1104, PMID: 30503099

[62] World Bank. World Bank Country and Lending Groups. (n.d.)Available at: https://datahelpdesk.worldbank.org/knowledgebase/articles/906519-world-bank-country-and-lending-groups (Accessed 25 July 2023).

