# Supplementary material for: Prevalence and correlates of subjective cognitive impairment in Chinese psychiatric patients during the fifth wave of COVID-19 in Hong Kong
Source: Front Psychiatry. 2023 Aug 17;14:1216768. doi: 10.3389/fpsyt.2023.1216768 (PMC10469870; doi:10.3389/fpsyt.2023.1216768)
Supplement: Supplementary file 1 [file Table_1.DOCX]

**Supplementary Table S1** Description of questionnaire items specifically designed for COVID-19 related factors.

| Variables | Description |
| --- | --- |
| Contracting the COVID-19 infection | Participants were first asked whether or not they had contracted COVID-19 before. If participants chose “yes”, they were asked if the latest infection was in “Dec 2021 or before” or “Jan 2022” or after (during fifth wave of COVID-19 outbreak). |
| Fear of contagion | Participants were asked to what extent they were fear of contracting COVID-19 using a 11-point Likert scale (0=Not afraid at all, 10=Extremely afraid). |
| COVID-19 vaccine doses received | Participants were asked how many doses of COVID-19 vaccination they have received by choosing “0” or “1” or “2” or “3”. |
| Time spent on reading COVID-19 related information | Participants were asked how much they have spent on reading COVID-19 related information on average each day in the past 2 weeks. “None”, “Less than an hour”, “1-3 hours”, “4-6 hours”, “More than 6 hours” were the options. |
| Number of COVID-19 stressors | Participants were asked the amount of stress they experienced during fifth wave in each of the listed aspects respectively. Eight aspects were assessed in total using a 5-point Likert scale ranging from 0 (not stressed) to 4 (extremely stressed): (1) financial, (2) work, (3) physical health, (4) mental health, (5) food and supplies, (6) medicine, (7) family relationship, (8) Other interpersonal relationships. A rating of 2 or above would be regarded as a stressor. |
| Experiences of being imposed on mandatory infection control measures | Participants were asked if they have experienced the following measures during fifth wave of COVID-19. Two measures were selected using a dichotomous (yes-no) scale: (1) Required to undergo mandatory quarantine without being tested positive and (2) Required to undergo mandatory testing as the building you lived in was listed in Restriction-testing Declaration |
| Distress from experiencing the tightening of social-distancing measures^a^ | Participants were asked to indicate their level of distress from experiencing the tightening of social distancing measures during fifth wave of COVID-19. A 11-point Likert scale (0=Not stressed at all, 10=Extremely stressed) was used. |

*COVID-19,* Coronavirus disease 2019

^a^ Tightening of social-distancing measures included prohibition of group gathering of more than 2 people in public place, restriction of no more than 2 customers per table in catering premises, dine-in ban after 6 p.m. and closure of all recreational premises.
